# Nucleated red blood cells, critical illness survivors and postdischarge outcomes: a cohort study

**DOI:** 10.1186/s13054-017-1724-z

**Published:** 2017-06-21

**Authors:** Steven W. Purtle, Clare M. Horkan, Takuhiro Moromizato, Fiona K. Gibbons, Kenneth B. Christopher

**Affiliations:** 1 Division of Pulmonary Sciences and Critical Care Medicine, University of Colorado, Boulder, CO USA; 2 Department of Medicine, Brigham and Women’s Hospital, Boston, MA USA; 3 Renal and Rheumatology Division, Internal Medicine Department, Okinawa Southern Medical Center and Children’s Hospital, Haebaru, Okinawa Japan; 4 Division of Pulmonary and Critical Care Medicine, Massachusetts General Hospital, Boston, MA USA; 5 The Nathan E. Hellman Memorial Laboratory, Renal Division, Channing Division of Network Medicine, Brigham and Women’s Hospital, MRB 418, 75 Francis Street, Boston, MA 02115 USA

**Keywords:** Nucleated red blood cells, Critical care, Mortality, Outcomes, Hospital readmission

## Abstract

**Background:**

Little is known about risk factors associated with out-of-hospital outcomes in survivors of critical illness. We hypothesized that the presence of nucleated red blood cells in patients who survived critical care would be associated with adverse outcomes following hospital discharge.

**Methods:**

We performed a two-center observational cohort study of patients treated in medical and surgical intensive care units in Boston, Massachusetts. All data were obtained from the Research Patient Data Registry at Partners HealthCare. We studied 2878 patients, age ≥ 18 years, who received critical care between 2011 and 2015 and survived hospitalization. The exposure of interest was nucleated red blood cells occurring from 2 days prior to 7 days after critical care initiation. The primary outcome was mortality in the 90 days following hospital discharge. Secondary outcome was unplanned 30-day hospital readmission. Adjusted odds ratios were estimated by multivariable logistic regression models with inclusion of covariate terms thought to plausibly interact with both nucleated red blood cells and outcome. Adjustment included age, race (white versus nonwhite), gender, Deyo–Charlson Index, patient type (medical versus surgical), sepsis and acute organ failure.

**Results:**

In patients who received critical care and survived hospitalization, the absolute risk of 90-day postdischarge mortality was 5.9%, 11.7%, 15.8% and 21.9% in patients with 0/μl, 1–100/μl, 101–200/μl and more than 200/μl nucleated red blood cells respectively. Nucleated red blood cells were a robust predictor of postdischarge mortality and remained so following multivariable adjustment. The fully adjusted odds of 90-day postdischarge mortality in patients with 1–100/μl, 101–200/μl and more than 200/μl nucleated red blood cells were 1.77 (95% CI, 1.23–2.54), 2.51 (95% CI, 1.36–4.62) and 3.72 (95% CI, 2.16–6.39) respectively, relative to patients without nucleated red blood cells. Further, the presence of nucleated red blood cells is a significant predictor of the odds of unplanned 30-day hospital readmission.

**Conclusion:**

In critically ill patients who survive hospitalization, the presence of nucleated red blood cells is a robust predictor of postdischarge mortality and unplanned hospital readmission.

**Electronic supplementary material:**

The online version of this article (doi:10.1186/s13054-017-1724-z) contains supplementary material, which is available to authorized users.

## Background

Nucleated red blood cells (NRBCs) are early erythrocyte precursors not present in the peripheral blood of normal adults. Fenestrations in the bone marrow provide a physical filter to the release of the large NRBCs into the circulation, and the rare NRBC that escapes is rapidly cleared from peripheral blood by the spleen [1]. The presence of circulating NRBCs in adults thus reflects extreme increases in erythropoietic activity or failure of the blood filtration mechanisms [2]. NRBCs are observed in peripheral blood in situations of hematopoietic stress such as inflammation, massive hemorrhage hematological malignancy, extramedullary hematopoiesis or severe hypoxia [3]. NRBCs have been shown to be present in approximately 10–30% of critically ill patients [4–8]. Although the mechanism for NRBC production in critical illness is not clear, the presence of NRBCs in the critically ill is associated with increased inhospital mortality [4–13].

With improvements in survival in those receiving critical care, the identification of risk factors for adverse out-of-hospital outcomes has gained importance [14–16]. ICU patients who survive to hospital discharge have substantial long-term morbidity, mortality and health care costs [17, 18]. Approximately 12% of ICU survivors are readmitted to the hospital following hospital discharge within 30 days [14]. Nearly 15% of ICU survivors die within 6 months of hospital discharge [19]. The risk factors for posthospital mortality in critical illness survivors are not well known. Tests or models that can predict postdischarge outcomes may be useful for targeting interventions in ICU survivors aimed at improving outcomes.

Although short-term survival has been explored in critically ill patients with NRBCs, postdischarge outcomes among ICU survivors with NRBCs is not known. NRBCs at the time of critical care may be a marker for critical illness survivors who are at high risk for subsequent adverse events. Given the heightened inhospital mortality in critically ill patients with NRBCs [4–7, 9–13], we sought to determine whether critically ill patients who develop NRBCs have an increased 90-day mortality following hospital discharge. We hypothesized that patients with NRBCs who survived critical care would have increased risk of postdischarge mortality.

## Methods

### Source population

We abstracted laboratory and administrative data from the electronic medical records of two teaching hospitals in Boston, Massachusetts: Brigham and Women’s Hospital (BWH), with 793 beds; and Massachusetts General Hospital (MGH), with 999 beds. Each hospital has approximately 45,000 hospital admissions per year.

### Data sources

Data on critically ill patients were collected prospectively in a central computerized registry called the Research Patient Data Registry (RPDR) [20] that serves as a central clinical data warehouse for all inpatient and outpatient records at Partners HealthCare sites including BWH and MGH. The RDPR has been used for other clinical research studies and mortality and coding data from the RPDR have been validated [21].

**Fig. 1:**
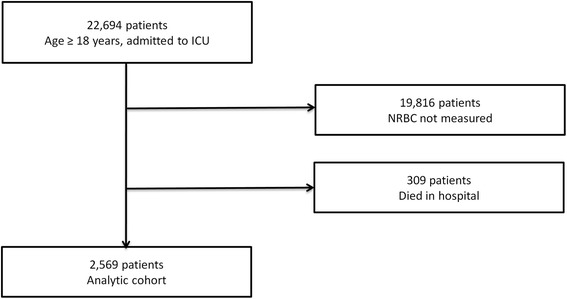
Flow chart for selection of study participants. *NRBC* nucleated red blood cells

Between 2011 and 2015, there were 22,694 unique patients, age ≥ 18 years, assigned Current Procedural Terminology (CPT) code 99291 (critical care, first 30–74 minutes) [21] at least twice and had a diagnosis-related group (DRG) assigned following hospitalization. A total of 19,816 patients did not have NRBCs measured from 2 days prior to 7 days after critical care initiation. Thus, 2878 patients constituted the parent cohort. A total of 309 parent cohort patients who died in the hospital were excluded. In total, 2569 patients constituted the analytic cohort (Fig. 1).

### Exposure of interest and comorbidities

The exposure of interest was the highest absolute NRBC count occurring from 2 days prior to 7 days after critical care initiation. The absolute NRBC count was determined via fluorescent flow cytometry using the Sysmex XE-5000 Automated Hematology System. The absolute NRBC count was reported as the number of NRBCs per microliter of blood [22], and was stratified as 0/μl, 1–100/μl, 101–200/μl and more than 200/μl [4].

Race was either self-determined or designated by a patient representative/healthcare proxy. We utilized the Deyo–Charlson Index to assess the burden of chronic illness, which is well studied and validated [23]. Patient type was defined as medical or surgical and incorporates the DRG methodology devised by Centers for Medicare & Medicaid Services [24]. We employed the validated International Classification of Diseases, Ninth Revision, Clinical Modification (ICD-9-CM) coding algorithms developed by Quan et al. [25] to derive a Deyo–Charlson Index comorbidity score for each patient [26].

Sepsis was defined by ICD-9 codes 038, 995.91, 995.92 or 785.52, from 3 days prior to critical care initiation to 7 days after critical care initiation [27]. The number of organs with failure was adapted from Martin et al. [28] and was defined by a combination of ICD-9-CM and CPT codes relating to acute organ dysfunction assigned from 3 days prior to critical care initiation to 30 days after critical care initiation [29, 30]. Noncardiogenic acute respiratory failure was identified by the presence of ICD-9 codes for respiratory failure or pulmonary edema (518.4, 518.5, 518.81, and 518.82) and mechanical ventilation (96.7×), excluding congestive heart failure (428.0–428.9) following hospital admission [31]. Pneumonia was identified by the presence of ICD-9 codes 480–486, from 3 days prior to 7 days after ICU admission [32]. Chronic kidney disease stage was determined by the Modification of Diet in Renal Disease (MDRD) equation from the baseline creatinine, age, gender and race of cohort patients [33]. The Acute Organ Failure score is an ICU risk-prediction score derived and validated from demographics (age, race), patient admission ‘type’ as well as ICD-9-CM code-based comorbidity, sepsis and acute organ failure covariates which has similar discrimination for 30-day mortality as Acute Physiology and Chronic Health Evaluation (APACHE) II [34]. Red blood cell transfusions were determined from blood bank records for the number of units of packed red blood cells transfused in the 7 days prior to the absolute NRBC count measurement. Recognizing that prior hospitalizations are important drivers of hospital readmission, hospitalization in the 2 years prior was determined by administrative data from BWH and MGH [35, 36].

### End points

The primary outcome was 90-day postdischarge mortality. Secondary outcomes included 30-day postdischarge mortality and unplanned 30-day hospital readmission. Information on vital status for the study cohort was obtained from the Social Security Administration Death Master File which we have validated for inhospital and out-of-hospital mortality in our administrative database [21]. One hundred percent of the cohort had vital status present at 90 days following critical care initiation. The censoring date was May 25, 2015. Thirty-day hospital readmission was determined from RPDR hospital admission data as described previously [14] and was defined as a subsequent or unscheduled admission to BWH or MGH within 30 days of discharge following the hospitalization associated with the critical care exposure [14, 37, 38]. We excluded readmissions with DRG codes that are commonly associated with planned readmissions in addition to DRG codes for transplantation, procedures related to pregnancy and psychiatric issues [14, 39].

### Power calculations and statistical analysis

Based on prior studies [14–16] we assumed that 90-day postdischarge hospital mortality would increase an absolute 7.5% in patients with NRBCs (15%) compared with those without NRBCs (7.5%). With an alpha error level of 5% and a power of 80%, the minimum sample size thus required for our primary end point is 608 total patients.

Categorical covariates were described by frequency distribution, and compared across NRBC groups using contingency tables and chi-square testing. Continuous covariates were examined graphically and in terms of summary statistics, and were compared across exposure groups using one-way ANOVA. Unadjusted associations between NRBC groups and outcomes were estimated by bivariable logistic regression analysis. Adjusted odds ratios were estimated by multivariable logistic regression models with inclusion of covariate terms thought to plausibly interact with both NRBCs and postdischarge hospital mortality.

Overall model fit was assessed using the Hosmer–Lemeshow test. Analyses based on fully adjusted models were performed to evaluate the NRBC–mortality association, and the *P* value for interaction was determined to explore for any evidence of effect modification. To evaluate for multicollinearity, we calculated the variance inflation factors and tolerances for each of the independent variables. Locally weighted scatter plot smoothing (LOWESS) was used to graphically represent the relationship between absolute NRBC count and rate of 90-day postdischarge mortality. Further, a multivariable Cox proportional hazards model was used to illustrate post-ICU admission and posthospital survival. All *P* values presented are two-tailed; values below 0.05 were considered nominally significant. All analyses are performed using STATA 13.1MP (College Station, TX, USA).

## Results

Patient characteristics of the parent cohort were stratified according to inhospital mortality (Table 1). In the parent cohort, age, surgical patients, Deyo–Charlson index, sepsis, pneumonia, acute respiratory failure, acute organ failure and NRBCs were significantly associated with inhospital mortality. In the study cohort of patients who survived hospitalization, the mean (SD) age at hospital admission was 60.7 (18.0) years. Most patients were male (54.1%), white (79.1%) and had a medically related DRG (55.4%). In total, 34.9% had a hospital admission in the 2 years prior, 43.5% were discharged to a care facility and 1.91% of patients were discharged to hospice care. In the study cohort, the 30-day, 90-day and 365-day postdischarge mortality rates were 4.7%, 8.1%, and 14.4%, respectively. The unplanned 30-day postdischarge hospital readmission rate was 12.1%. Factors that were associated with stratified NRBC category included higher age, patient type, Deyo–Charlson Index, sepsis, pneumonia, acute respiratory failure, acute organ failure and leukemia or myelodysplastic syndrome (Table 2). Patients who did not have NRBCs measured in the time period of interest had similar demographics, higher acute organ failures, sepsis and pneumonia, and similar mortality rates to those patients with NRBCs measured (Additional file 1).

**Table 1:** Clinical and demographic characteristics of the parent cohort (*n* = 2878)

	Alive at discharge	Expired	Total	*P* value (chi-square test)	Unadjusted OR (95% CI) for inhospital mortality
*N*	2569	309	2878		
Male gender	1390 (54)	178 (58)	1568 (54)	0.24	1.15 (0.91–1.46)
Nonwhite race	536 (21)	66 (21)	602 (21)	0.84	1.03 (0.77–1.37)
Age (years)	60.7 *±* 18.0	67.0 *±* 16.8	61.4 *±* 18.0	<0.001*	1.02 (1.01–1.03)
Surgical patient type	1147 (45)	92 (30)	1239 (43)	<0.001	0.53 (0.41–0.68)
Deyo–Charlson Index				<0.001	
0	533 (21)	29 (9)	562 (20)		1.00 (Referent)
1–2	628 (24)	54 (17)	682 (24)		1.58 (0.99–2.52)
3–6	566 (22)	69 (22)	635 (22)		2.24 (1.43–3.51)
≥7	842 (33)	157 (51)	999 (35)		3.43 (2.27–5.17)
Sepsis	241 (9)	113 (37)	354 (12)	<0.001	
Pneumonia	487 (19)	106 (34)	593 (21)	<0.001	2.23 (1.73–2.88)
Noncardiac acute respiratory failure	169 (7)	85 (28)	254 (9)	<0.001	5.39 (4.01–7.23)
Acute organ failure				<0.001	
0	843 (33)	5 (2)	848 (29)		1.00 (Referent)
1	895 (35)	65 (21)	960 (33)		12.25 (4.91–30.56)
2	508 (20)	73 (24)	581 (20)		24.23 (9.73–60.35)
≥3	323 (13)	166 (54)	489 (17)		86.65 (35.27–212.87)
Leukemia or myelodysplastic syndrome	229 (9)	53 (17)	282 (10)	<0.001	2.12 (1.53–2.93)
Hematocrit	34.1 ± 7.0	30.9 ± 7.0	33.8 ± 7.1	<0.001*	0.94 (0.92–0.95)
Red cell distribution width	14.8 ± 2.2	16.9 ± 2.9	15.1 ± 2.4	<0.001*	1.32 (1.27–1.38)
Chronic kidney disease	800 (31)	175 (57)	975 (34)	<0.001	2.89 (2.27–3.67)
Acute Organ Failure score	10.0 ± 4.7	15.6 ± 5.0	10.6 ± 5.0	<0.001*	1.26 (1.23–1.30)
NRBCs				<0.001	
0	1867 (73)	101 (33)	1968 (68)		1.00 (Referent)
1–99	487 (19)	83 (27)	570 (20)		3.15 (2.32–4.28)
100–199	101 (4)	43 (14)	144 (5)		7.87 (5.23–11.85)
≥200	114 (4)	82 (27)	196 (7)		13.30 (9.39–18.82)
Mortality rate (%)					
Inhospital			309 (10.7)		
30-day			392 (13.6)		
90-day			509 (17.7)		

Data presented as *n* (%) or mean **±** SD

*P value determined by ANOVA

*CI* confidence interval, *NRBC* nucleated red blood cell, *OR* odds ratio

**Table 2:** Characteristics of the study cohort stratified by NRBCs (*n* = 2569)

	0/μl	1-100/μl	101-200/μl	>200/μl	*P* value (chi-square test)
*N*	1867	487	101	114	
Male gender	1025 (55)	248 (51)	60 (59)	57 (50)	0.22
Nonwhite race	380 (20)	109 (22)	20 (20)	27 (24)	0.66
Age (years)	60.1 *±* 18.5	62.7 *±* 16.4	63.2 *±* 15.4	61.4 *±* 17.4	0.014^*^
Surgical patient type	785 (42)	253 (52)	55 (54)	54 (47)	<0.001
Deyo–Charlson Index					<0.001
0	444 (24)	63 (13)	9 (9)	17 (15)	
1–2	473 (25)	111 (23)	21 (21)	23 (20)	
3–6	394 (21)	113 (23)	30 (30)	29 (25)	
≥7	556 (30)	200 (41)	41 (41)	45 (40)	
Sepsis	118 (6)	77 (16)	22 (22)	24 (21)	<0.001
Pneumonia	293 (16)	125 (26)	31 (31)	38 (33)	<0.001
Noncardiac acute respiratory failure	98 (5)	45 (9)	13 (13)	13 (11)	<0.001
Acute organ failure					<0.001
0	721 (39)	94 (19)	10 (10)	18 (16)	
1	663 (36)	169 (35)	37 (37)	26 (23)	
2	337 (18)	118 (24)	25 (25)	28 (25)	
≥3	146 (8)	106 (22)	29 (29)	42 (37)	
Leukemia or myelodysplastic syndrome	131 (7)	55 (11)	19 (19)	24 (21)	<0.001
Hematocrit	35.5 *±* 6.6	31.2 *±* 6.6	29.2 ± 7.4	28.8 ± 7.0	<0.001
Red cell distribution width	13.9 (13.1–15.1)	15.3 (14.2–17.1)	16.3 (15.1–18.0)	17.1 (15.5–19.3)	
Chronic kidney disease	510 (27)	197 (40)	39 (39)	54 (47)	<0.001
Transfusions	0 (0–0)	0 (0–1)	1 (0–3)	0 (0–2)	<0.001^†^
Acute Organ Failure score	9.5 ***±*** 4.4	10.9 ***±*** 4.9	11.9 ***±*** 5.1	12.0 ***±*** 5.9	<0.001^*^
NRBCs	0 (0–0)	40 (20–60)	140 (120–170)	375 (270–690)	<0.001^†^
Postdischarge mortality rate (%)					
30-day	58 (3.1)	34 (7.0)	12 (11.9)	17 (14.9)	<0.001
90-day	111 (6.0)	57 (11.7)	16 (15.8)	25 (21.9)	<0.001

Data presented as *n* (%), mean **±** SD, or median (interquartile range)

*P value determined by ANOVA

^**†**^ *P* value determined by Kruskal–Wallis test

*NRBC* nucleated red blood cell

### Primary outcome

**Fig. 2:**
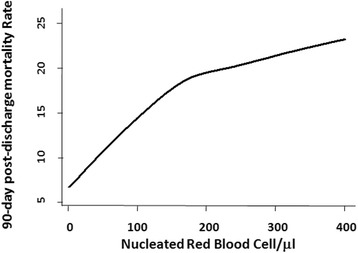
NRBC status versus 90-day postdischarge mortality rate. Locally weighted scatter plot smoothing (LOWESS) utilized to represent the near inverse linear association between absolute NRBC count and 90-day postdischarge mortality rate. With bandwidth parameter = 0.99; 2075 cohort patients were utilized to construct the curve

Mortality risk in the 90 days after hospital discharge was higher in patients with increasing NRBCs (Fig. 2). The odds of 90-day postdischarge mortality in patients with 1–100/μl, 101–200/μl and more than 200/μl NRBCs were 2.1-fold, 3.0-fold and 4.4-fold higher respectively than patients with 0/μl NRBCs (Table 3). The NRBC level remained a significant predictor of the odds of 90-day postdischarge mortality after adjustment for age, gender, race, Deyo–Charlson index, patient type, sepsis and acute organ failure. The adjusted odds of 90-day postdischarge mortality in patients with 1–100/μl, 101–200/μl and more than 200/μl NRBCs were 1.8-fold, 2.5-fold and 3.7-fold higher respectively than patients with 0/μl NRBCs (Table 3). The adjusted 90-day postdischarge mortality model showed good calibration (Hosmer–Lemeshow χ^2^ = 6.66, *P* = 0.57) and discrimination (*c*-statistic = 0.80 (95% CI 0.77–0.82)) and there was no multicollinearity as determined by the variance inflation factor. Analyzing the adjusted model with and without the NRBC term shows significantly improved discrimination for 90-day postdischarge mortality with NRBCs included (χ^2^ = 6.23, *P* = 0.013). Further, the hazard ratio of postdischarge mortality adjusted for age, gender, race, Deyo–Charlson index, patient type, sepsis and acute organ failure in patients with 1–100/μl, 101–200/μl and more than 200/μl NRBCs were 1.57 (95% CI 1.26–1.96), 1.97 (95% CI 1.35–2.90) and 3.02 (95% CI 2.15–4.24) respectively relative to patients with 0/μl NRBCs. Compared with the analytic cohort, the hazard ratio of post-ICU admission mortality (*N* = 2878) adjusted for age, gender, race, Deyo–Charlson index, patient type, sepsis and acute organ failure in patients with 1–100/μl, 101–200/μl and more than 200/μl NRBCs were 1.60 (95% CI 1.33–1.92), 2.35 (95% CI 1.80–3.08) and 3.47 (95% CI 2.75–4.37) respectively relative to patients with 0/μl NRBCs.

**Table 3:** Unadjusted and adjusted associations between nucleated red blood cells and mortality (*n* = 2569)

	Mortality odds ratio (95% CI)^a^			
	0/μl NRBCs	1–100/μl NRBCs	101–200/μl NRBCs	>200/μl NRBCs
30-day post discharge mortality				
Crude	1.00 (Referent)	2.34 (1.51—3.62)	4.21 (2.18—8.11)	5.47 (3.07—9.74)
Adjusted^b^	1.00 (Referent)	2.02 (1.27—3.21)	3.66 (1.81—7.39)	4.64 (2.45—8.79)
Adjusted^c^	1.00 (Referent)	1.85 (1.16—2.95)	3.06 (1.50—6.22)	3.51 (1.82—6.77)
Adjusted^d^	1.00 (Referent)	1.86 (1.19—2.90)	2.90 (1.46—5.74)	3.66 (1.98—6.75)
90-day post discharge mortality				
Crude	1.00 (Referent)	2.10 (1.50—2.94)	2.98 (1.69—5.25)	4.44 (2.74—7.21)
Adjusted^b^	1.00 (Referent)	1.77 (1.23—2.54)	2.51 (1.36—4.62)	3.72 (2.16—6.39)
Adjusted^c^	1.00 (Referent)	1.67 (1.16—2.41)	2.19 (1.18—4.05)	3.05 (1.75—5.34)
Adjusted^d^	1.00 (Referent)	1.65 (1.16—2.34)	2.03 (1.12—3.69)	2.99 (1.78—5.04)

*CI* confidence interval, *NRBC* nucleated red blood cell

^a^Referent in each case is absence of NRBCs

^b^Model 1: estimates adjusted for age, gender, race, Deyo–Charlson index, type (surgical vs medical), sepsis and acute organ failure

^c^Model 2: estimates adjusted for age, gender, race, Deyo-Charlson index, type (surgical vs medical), sepsis, acute organ failure, hematocrit < 30, leukemia or myelodysplastic syndrome

^d^Model 3: estimates adjusted for gender and the Acute Organ Failure score

There was no significant effect modification of the NRBC-90-day postdischarge mortality association on the basis of sepsis (*P*-interaction = 0.14), hospital (*P*-interaction = 0.87), hematocrit (*P*-interaction = 0.52) or pneumonia (*P*-interaction = 0.053). Effect modification is present regarding the presence of leukemia or myelodysplastic syndrome (*P*-interaction = 0.025) and RDW (P-interaction = 0.001). Individually adding a leukemia or myelodysplastic syndrome or Red blood cell distribution width (RDW) term to the final model does not alter the effect size or significance of the change in NRBC–90-day postdischarge mortality association (data not shown). While patients with and without elevated RDW present have different risk estimates, the directionality and significance of the NRBC–postdischarge mortality association is unchanged. Further in the analytic cohort, the RDW-NRBC Pearson's correlation coefficient = 0.16 (*P* < 0.001), indicative of a very small but significant association. Finally, when patients with leukemia or myelodysplastic syndrome (*n* = 229) were excluded, the adjusted odds of 90-day postdischarge mortality in patients with NRBCs present was 2.1-fold higher respectively than patients without NRBCs (OR = 2.07 (95% CI 1.43–3.00); *P* < 0.001).

The presence of NRBCs was a strong predictor of 30-day hospital readmission. The odds of 30-day hospital readmission in patients with NRBCs present was 1.8-fold that of patients without NRBCs (OR = 1.84 (95% CI 1.44–2.35); *P* < 0.001). The presence of NRBCs remained a significant predictor of the odds of 30-day hospital readmission after adjustment for age, sex, race, Deyo–Charlson Index, patient type, acute organ failure and sepsis. The adjusted odds of 30-day hospital readmission in patients with NRBCs present was 1.6-fold that of patients without NRBCs (OR = 1.57 (95% CI 1.20–2.04; *P* = 0.001). When we add a term for previous hospitalization in the prior 2 years, the adjusted odds of 30-day hospital readmission in patients with NRBCs present was 1.6-fold that of patients without NRBCs (OR = 1.55 (95% CI 1.19–2.02; *P* = 0.001).

### Validation

To validate out-of-hospital mortality, we considered hospital mortality and outpatient encounter data from the RPDR [20] and Master Death File data from the Social Security Administration [40]. Patients were considered alive if an inpatient or outpatient encounter was recorded later than 90 days following hospital discharge. Patients were considered deceased if hospital mortality discharge disposition was noted as diseased. In the 1925 cohort patients in whom hospital mortality or outpatient encounter data existed, the sensitivity and specificity of the Master Death File for RPDR recorded 90-day postdischarge mortality were 99% and 97% respectively.

## Discussion

ICU survivors are known to have substantial long-term morbidity and mortality [17, 19]. Risk factors for adverse events following ICU discharge include comorbidity, severity of illness, organ failure indices, high ICU occupancy, ICU discharge time and facility type where discharged [41–44]. The risk factors for posthospital death in critical illness survivors are not well known. In this study, we investigated whether the presence of peripheral NRBCs in survivors of critical illness was associated with the risk of postdischarge outcomes. Our novel observations demonstrate that the presence of NRBCs is associated with a significant increase in the odds of postdischarge hospital mortality. While causation cannot be inferred from an observational study, the NRBC–mortality association has biologic plausibility. The appearance of NRBCs in the circulation implies both increased erythropoietic pressure and failure of the spleen to remove these abnormal cells from the circulation. Because conditions like systemic inflammation, hypoxia or massive hemorrhage are known to increase erythropoietic pressure, the appearance of NRBCs in peripheral blood signals a disturbance in physiologic homeostasis. Hematologic parameters such as the red cell distribution width and NRBC count may therefore be markers for acute organ failure in the same way that serum creatinine or liver transaminases are markers for renal and hepatic dysfunction, respectively.

Several of the critical care severity of illness scoring systems incorporate abnormalities in the complete blood count, but erythrocyte parameters are relatively underrepresented. The APACHE II system incorporates values of the white blood cell count and hematocrit. The Simplified Acute Physiology Score (SAPS) II utilizes only the white blood cell count, and the Multiorgan Dysfunction Syndrome (MODS) score takes only platelets into account. The ability to accurately stratify a homogeneous population of critically ill patients is important for large clinical trials in the ICU, where the effect of an intervention may be masked by heterogeneity in the severity of illness among treatment arms. Based on the robust association between NRBCs and mortality, it is possible that the addition of red blood cell indices, such as the NRBC count or red cell distribution width, could improve the accuracy of future iterations of these scoring systems. The presence of NRBCs in our study appears to be a marker of severity of illness and not a marker that is specific to posthospital outcomes per se. Beyond the prognostication of postdischarge mortality, we also demonstrate that patients with NRBCs have increased risk of hospital readmission following discharge which is of a clinically significant magnitude in light of the high priority to reduce readmission among Medicare patients [45].

The present study may have limitations. Postdischarge outcomes may be influenced by other unmeasured variables independently of NRBCs, which could bias estimates. Ascertainment bias may be present because not all critically ill patients have NRBCs measured because it is included in the white blood cell differential. Reliance on ICD-9 codes to determine comorbidities will underestimate the true incidence, which is likely higher [46]. Despite adjustment for multiple potential confounders, residual confounding may be present contributing to observed differences in outcomes. We are unable to adjust for physiologic-based severity of illness scores which are strong predictors of critical illness outcome [47]. We have adjusted for an ICU risk prediction score validated against APACHE II [34] but it is conceivable that inclusion of a physiologic score in the analysis may materially alter the NRBC–postdischarge mortality association. However, despite multivariable adjustment, the absence of physiologic data is a potential limitation of our study. Further, our readmission data include only hospital readmissions from the two institutions under study, which may have underestimated the total readmission rates.

Our study has several strengths. We have validated the use of CPT code 99291 in a prior study to identify patients in the RDPR dataset who are admitted to an ICU [21]. The 30-day time point for hospital readmission is frequently utilized in outcome research [48–51], and is the statistically optimal approach for readmission rates [52, 53]. From our validation data, it appears that the Master Death File accurately captures postdischarge mortality in our population.

## Conclusion

On aggregate, these data demonstrate that NRBCs are associated with increased postdischarge mortality and hospital readmission. The identification of exposures that are predictive of out-of-hospital outcomes may be useful for targeted interventions. If our observations are corroborated in other cohorts, ICU patients with NRBCs who survive to discharge might benefit from a more intense follow-up schedule and enhanced longitudinal care.

## Additional files

Additional file 1: Table presenting clinical and demographic characteristics of the parent cohort (*n* = 22,694). (PDF 64 kb)

## Abbreviations

APACHE: Acute Physiology and Chronic Health Evaluation
BWH: Brigham and Women’s Hospital
CPT: Current Procedural Terminology
DRG: Diagnosis-related group
ICD-9-CM: International Classification of Diseases, Ninth Revision, Clinical Modification
ICU: Intensive care unit
LOWESS: Locally weighted scatter plot smoothing
MDRD: Modification of Diet in Renal Disease
MGH: Massachusetts General Hospital
MODS: Multiorgan Dysfunction Syndrome
NRBC: Nucleated red blood cell
RPDR: Research Patient Data Registry
SAPS: Simplified Acute Physiology Score
