# Supplementary material for: Nucleated red blood cells, critical illness survivors and postdischarge outcomes: a cohort study
Source: Crit Care. 2017 Jun 21;21:154. doi: 10.1186/s13054-017-1724-z (PMC5479031; doi:10.1186/s13054-017-1724-z)
Supplement: Supplementary file 1 — Table presenting clinical and demographic characteristics of the parent cohort (n = 22,694). (PDF 64 kb) [file 13054_2017_1724_MOESM1_ESM.pdf]

## Additional file 1: Clinical and demographic characteristics of the parent cohort (n=22,694)

| | no.(%) without NRBC | no.(%) with NRBC | Total | P-value |
|---|---|---|---|---|
| *N* | **19,816** | **2,878** | **22,694** | |
| *Male Gender-no.(%)* | **11,475 (58)** | **1,568 (55)** | **13,043 (57)** | **0.001** |
| *Non-White Race-no.(%)* | **4,056 (20)** | **601 (21)** | **4,657 (21)** | **0.60** |
| *Age years-mean±SD* | **62.0 ± 18.9** | **61.4±18.0** | **62.0±18.8** | **0.093** |
| *Surgical Patient Type-no.(%)* | **8,155 (41)** | **1,239 (43)** | **9,394 (41)** | **0.051** |
| *Deyo-Charlson Index-no.(%)* | | | | **<0.001** |
| 0 | **3305 (17)** | **562 (20)** | **3,867 (17)** | |
| 1-2 | **5258 (27)** | **682 (24)** | **5,940 (26)** | |
| 3-6 | **5181 (26)** | **635 (22)** | **5,816 (26)** | |
| ≥ 7 | **6073 (31)** | **999 (35)** | **7,071 (31)** | |
| *Sepsis-no.(%)* | **3,339 (17)** | **354 (12)** | **3,693 (16)** | **<0.001** |
| *Pneumonia-no.(%)* | **5,605 (28)** | **593 (21)** | **6,198 (27)** | **<0.001** |
| *Acute organ failure-no.(%)* | | | | **<0.001** |
| 0 | **4,077 (21)** | **848 (29)** | **4,925 (22)** | |
| 1 | **5,654 (29)** | **960 (33)** | **6,613 (29)** | |
| 2 | **4,561 (23)** | **581 (20)** | **5,142 (23)** | |
| ≥ 3 | **5,525 (28)** | **489 (17)** | **6,014 (27)** | |
| *Mortality Rates %* | | | | |
| In-hospital | **2,599 (13)** | **309 (11)** | **2,908 (13)** | **<0.001** |
| 30-day | **2,846 (14)** | **392 (14)** | **3,238 (14)** | **0.29** |
| 90-day | **3,617 (18)** | **509 (18)** | **4,126 (18)** | **0.47** |

Data presented as n (%) unless otherwise indicated. P values determined by chi-square unless designated by (*) then P value determined by ANOVA.
